# Comparison of Systolic Blood Pressure Values Obtained by Photoplethysmography and by Korotkoff Sounds

**DOI:** 10.3390/s131114797

**Published:** 2013-10-31

**Authors:** Meir Nitzan, Yair Adar, Ellie Hoffman, Eran Shalom, Shlomo Engelberg, Iddo Z. Ben-Dov, Michael Bursztyn

**Affiliations:** 1 Department of Physics/Electro-Optics, Jerusalem College of Technology, Jerusalem 9116001, Israel; E-Mails: yadar@g.jct.ac.il (Y.A.); hoffman18@gmail.com (E.H.); eransh@g.jct.ac.il (E.S.); 2 Department of Electronics, Jerusalem College of Technology, Jerusalem 9116001, Israel; E-Mail: shlomoe@jct.ac.il; 3 Nephrology and Hypertension Services, Hadassah-Hebrew University Medical Center, Jerusalem 9112000, Israel; E-Mail: iddo@hadassah.org.il; 4 Department of Internal Medicine, Hadassah-Hebrew University Medical Center, Jerusalem 9124000, Israel; E-Mail: bursz@mail.huji.ac.il

**Keywords:** systolic blood pressure, sphygmomanometry, Korotkoff sounds, photoplethysmography, accuracy

## Abstract

In the current study, a non-invasive technique for systolic blood pressure (SBP) measurement based on the detection of photoplethysmographic (PPG) pulses during pressure-cuff deflation was compared to sphygmomanometry—the Korotkoff sounds technique. The PPG pulses disappear for cuff-pressures above the SBP value and reappear when the cuff-pressure decreases below the SBP value. One hundred and twenty examinations were performed on forty subjects. In 97 examinations the two methods differed by less than 3 mmHg. In nine examinations the SBP value measured by PPG was higher than that measured by sphygmomanometry by 5 mmHg or more. In only one examination the former was lower by 5 mmHg or more than the latter. The appearance of either the PPG pulses or the Korotkoff sounds assures that the artery under the cuff is open during systolic peak pressure. In the nine examinations mentioned above the PPG pulses were observed while Korotkoff sounds were not detected, despite the open artery during systole. In these examinations, the PPG-based technique was more reliable than sphygmomanometry. The high signal-to-noise ratio of measured PPG pulses indicates that automatic measurement of the SBP by means of automatic detection of the PPG signals is feasible.

## 1. Introduction

### 1.1. Manual and Automatic Blood Pressure Measurement

The physiological and clinical significance of the systolic (SBP) and diastolic (DBP) arterial blood pressure has motivated many efforts to develop a reliable noninvasive blood pressure measurement technique. In sphygmomanometry (SPM), an external pressure cuff is used, and the commencement and the cessation of the Korotkoff sounds when the cuff pressure decreases below the SBP and DBP values, respectively, are identified. Auscultatory SPM uses audible detection of Korotkoff sounds, and, when performed by a trained listener, is considered to be the most accurate non-invasive method, and is accepted as a reference standard for evaluating the accuracy of other non-invasive methods for blood pressure measurement. However, the auscultatory SPM is not convenient to use, depends on the experience of the user and only provides single measurements. Furthermore, the presence of the physician can influence blood pressure level, which is known as the white coat effect [1–3].

In order to avoid the drawback of the auscultatory SPM, non-invasive automatic blood pressure (NIBP) meters have been developed. The automatic SPM detects the Korotkoff sounds by means of a microphone but is prone to artifacts due to external noise and vibrations [4,5]. Oscillometry, the most prevalent automatic NIBP technique, is based on the measurement of air pressure oscillations in the pressure cuff induced by the pulsating arteries during cuff deflation, after inflating the cuff air pressure to above the systolic blood pressure. SBP and DBP are derived from the envelope of the oscillometric curve using empirical criteria [5–7]. These empirical criteria are based on statistical considerations and are the main source of error in oscillometry, since the shape of the oscillometric curve envelope does not depend merely on the SBP and the DBP values, but also on the characteristics of the arteries under the cuff and the characteristics of the cuff itself [3,7–9]. It is important to note that the oscillometric pulses in the cuff pressure also appear for cuff pressure values above the SBP value, even though the arteries under the pressure cuff are closed, due to the impact of the arterial blood pulses on the proximal (upstream) end of the cuff. Most of the NIBP meters use oscillometry, automatic auscultation, or both [5].

The low accuracy of the available automatic NIBP meters can be deduced from the standards imposed by the Association for the Advancement of Medical Instrumentation [10], the protocol designed by the British Hypertension Society (BHS) [1] and the recommendations of the European Society of Hypertension (ESH) [11]. The standards are based on comparing the automated NIBP meter to manual SPM for three examinations performed on each member of a group of 85 or 33 subjects. [1,10–12]. The AAMI standard requires that the mean difference between the SBP (or DBP) values measured by the auscultatory SPM and the device under examination should not exceed 5 mmHg, and the standard deviation of that difference should not exceed 8 mmHg. According to these standards, a device is acceptable even if 5% of its examinations differ from those of the reference device by 16 mmHg or more. The recommendations of the BHS and ESH are similar. The reason for not demanding higher accuracy seems to be the low accuracy of the available devices that are generally based on the oscillometry method.

Nevertheless several recent Editorial Commentaries in *Hypertension* [13–15] emphasize the added value of home blood pressure measurements and ambulatory blood pressure monitoring. Increasing evidence indicates that information about blood pressure variability obtained by monitoring provides prognostic information regarding organ damage and cardiovascular events beyond that derived from the average blood pressure value, obtained in office measurements [14,15]. Some studies even show better prediction of cardiovascular events by single home blood pressure measurements than by office measurements [13].

### 1.2. SBP Measurement

Several techniques have been developed for the measurement of SBP by using the collapse of the artery under the cuff when the cuff pressure is above the SBP value. The reopening of the artery when the cuff pressure decreases below the SBP value can be detected by a distal flow or pulse detector, such as manual palpation, Doppler ultrasound, photoplethysmography (PPG) or strain gauge [16–25]. Each of these techniques uses the detection of a flow-related signal which starts to reappear when the cuff pressure decreases below the SBP value, and the arteries under the cuff reopen for a short time during the cardiac cycle. In contrast to oscillometry, these techniques (like the auscultatory SPM which is based on the detection of Korotkoff sounds), enable the measurement of SBP with no need for an empirical formula, that must be based on statistical grounds.

In a former study [24], we presented a cuff-based technique for automatic measurement of systolic blood pressure, based on PPG signals measured simultaneously in fingers of both hands. PPG is the measurement of light transmission changes during the cardiac cycle due to the arterial blood volume changes in the tissue, induced by heart activity. After inflating a pressure cuff to a level above the SBP value, it was slowly deflated, and the cuff pressure for which the PPG signal reappeared during the deflation of the pressure-cuff was taken as the SBP value. However the first PPG signals in several cases were small relative to the noise and their automatic detection required discrimination between true PPG pulses and random fluctuations. The algorithm for the selection of the PPG pulses was based on the values of two waveform parameters, calculated in the time-segments in which the PPG signals distal to the cuff were expected to appear, utilizing a reference PPG signal from the free hand. The detected pulses in these time-segments were identified as PPG pulses if they met two criteria that were based on the pulse waveform and on the correlation between the signal in the current segment and the signal in the two neighboring segments. The signal was taken as a PPG signal if minimal values of the two calculated pulse shape parameters were obtained in several time-segments. The minimal values and the number of time-segments were determined in order to achieve minimal standard deviation of the differences between the values of the SBP values, obtained empirically by the PPG-based automatic technique and auscultatory SPM, the reference standard.

That study considered the auscultatory method as the reference standard and tried to find another technique, with measurement results as close as possible to those of the reference standard. The determination of the pulses as PPG pulses was based on empirical criteria and was therefore of a statistical nature, which is a potential source of error, similar to oscillometry. Furthermore, the auscultatory technique has its own inaccuracies, and these inaccuracies are also involved in the PPG-based technique which is based on the auscultatory SPM.

In the current study, an improved noninvasive PPG-based method for the measurement of SBP is presented and compared to auscultatory SPM. The PPG signal downstream to the cuff disappears when the cuff pressure is greater than the SBP value and reappears when the cuff pressure decreases below the SBP value. In the current study the PPG signal-to-noise ratio was increased relative to the former study [24] and the detection was done visually off-line, by inspecting the light transmission curves during the deflation period. Only pulses that were certainly recognized as PPG pulses were accepted for the determination of the SBP. In general the signal-to-noise ratio of the PPG signal was high even at cuff pressures slightly below the SBP and the detection of the first PPG pulses was unequivocal. In the current study the comparison of the PPG-based method to the auscultatory SPM was not done in order to determine the accuracy of the former relative to the reference standard. In the following we show that in cases where the SBP measurements by the two techniques deviate, it is possible to determine which result is more accurate without the need to utilize invasive intra-arterial SBP measurement, the gold standard.

## 2. Experimental Section

### 2.1. Subjects and Method

PPG examinations and auscultatory SPM measurements were performed on 42 male subjects aged 25–73 years. We preferred to perform the examinations on male subjects, since in a previous study [26] we found that the PPG signal in female subjects is generally small relative to that of male subjects. Most of the examinees had no known cardiovascular disease, while others were diabetic or had cardiac diseases. 37 of the subjects were normotensive (SBP < 140 mmHg and DBP < 90 mmHg). All subjects had an arm circumference of 22–32 cm, compatible with the standard pressure-cuff that was used in the study. In two subjects an auscultatory gap of about 10 mmHg was found, and their examinations were not included in the current study.

**Figure 1.**
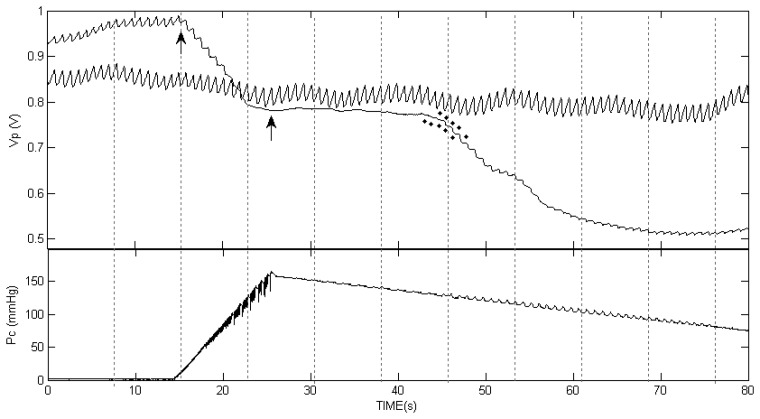
The PPG signals (V_P_) in the two hands of one of the subjects (above) and the cuff pressure (P_c_) as a function of time (below), during cuff inflation to above the SBP value and subsequent slow cuff deflation. The PPG pulses distal to the cuff disappeared when the cuff pressure increased to above the SBP value and reappeared when the cuff pressure decreased below the SBP value. The arrows indicate the start and stop of the cuff inflation. The stars above and below the PPG curve indicate the detection of the first Korotkoff sounds by the two listeners.

The subjects were seated during the examination. Their forearms were placed comfortably on a table, and the measurement was begun after a rest period of 10 min. A pressure cuff was applied to the left arm and an electronic pump raised the air pressure to 140, 160 or 180 mmHg (according to the SBP of the subject) at a rate of 15 mmHg per second. Then the air pressure decreased at a constant rate of 1.5 mmHg per second. The cuff air pressure was measured by a piezoelectric transducer which was calibrated by a mercury manometer. The SBP of each subject was measured five times, simultaneously by auscultatory SPM and by the PPG method, using the same pressure-cuff. Korotkoff sounds were detected by two researchers (MN and YA), using a double stethoscope. Each investigator pressed a button when the first 4–5 Korotkoff sounds were heard and each button-press caused a notation (star) to be inserted into the recording of the PPG (see Figure 1 in the Results Section).

The subjects were examined in the Jerusalem College of Technology. The study was approved by the institutional ethical committee of Hadassah–Hebrew University Medical Center, Jerusalem. All subjects signed an informed consent form.

### 2.2. The PPG-Based Device

Two reflection PPG probes, using infrared light sources were applied to the middle fingers of the two hands. The light source and the detector of each probe were placed on the two ipsilateral sides of the fingertip. This configuration was found to produce the highest signal-to-noise ratio. The PPG signals were sampled (at a rate of 1000 samples per second and with 16 bit resolution) and stored for further processing. The output of the piezoelectric transducer for the cuff air pressure measurement was simultaneously digitized and stored.

The PPG signal in the hand distal to the cuff was used for the determination of the cuff pressure at which the PPG pulses reappeared during cuff deflation. The PPG signal in the free hand was utilized for the determination of the time-segments in which the PPG signal distal to the cuff was expected to appear. In previous studies [24,27] we have found that the PPG signal distal to the cuff is expected to appear about 100–300 ms after the PPG signal in the free hand, when the cuff pressure is slightly below the SBP value. We used this information to validate that pulses in the finger distal to the cuff were actually PPG signals.

The increase of the cuff pressure was relatively slow, at a rate of 15 mmHg per second—it took more than 10 s to raise the pressure to a value of 160 mmHg. In an earlier study it was found that when the cuff is inflated at a higher rate, 10%–20% of subjects show no PPG pulses until the pressure has decreased significantly below the true SBP value (as measured by Korotkoff sounds). This effect probably originates from the collapse of the finger arteries under the PPG sensor due to their drainage into the veins when the cuff air pressure increases above SBP. When the cuff air pressure is slightly below the SBP value the blood volume pulses entering the arteries distal to the cuff are small and cannot open the collapsed arteries under the sensor [24]. Increasing the cuff pressure to above the SBP value slowly inhibits significant drainage of the blood from the arterial circulation and prevents the possible collapse of the small arteries under the PPG probe.

### 2.3. Derivation of SBP

Five examinations were taken for each subject. The first examination was only a practice run and was not used in the analysis. In general the next three examinations were analyzed for the determination of the SBP value; if one of these examinations could not be used because of movement noise, the fifth examination was used instead.

**Figure 2.**
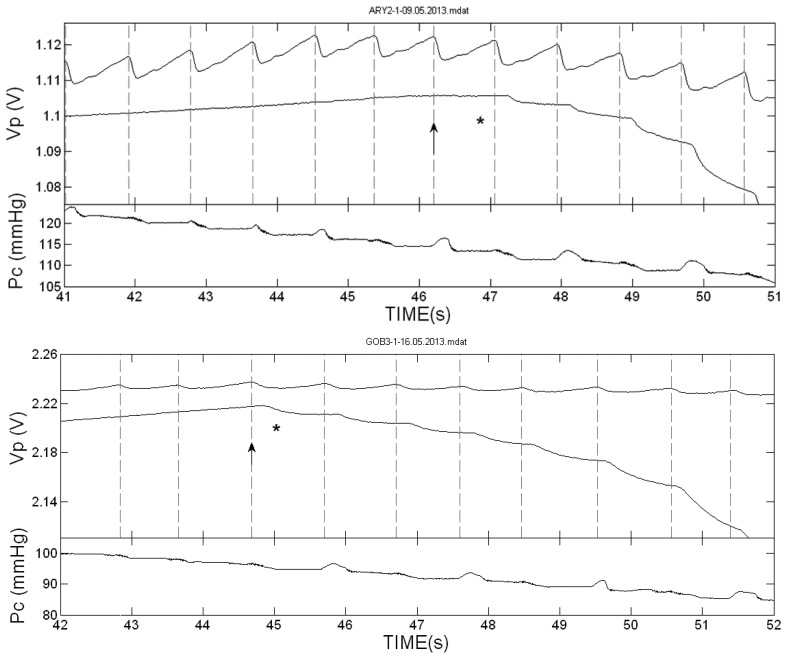
The PPG pulses (V_P_) in the two hands of two of the subjects during cuff deflation for cuff pressures in the neighborhood of the SBP value. The vertical dotted lines indicate the start time of the systolic decrease in the cuff-free hand PPG pulses (the upper curve in each figure). The star indicates the time of the first detection of the Korotkoff sounds and the arrow indicates the start time of the free-hand PPG-pulse in which the first star appeared. In these two examinations, the first Korotkoff sounds were heard in the same heart-beat or nearly the same heart beat in which the first PPG pulses were detected.

**Figure 3.**
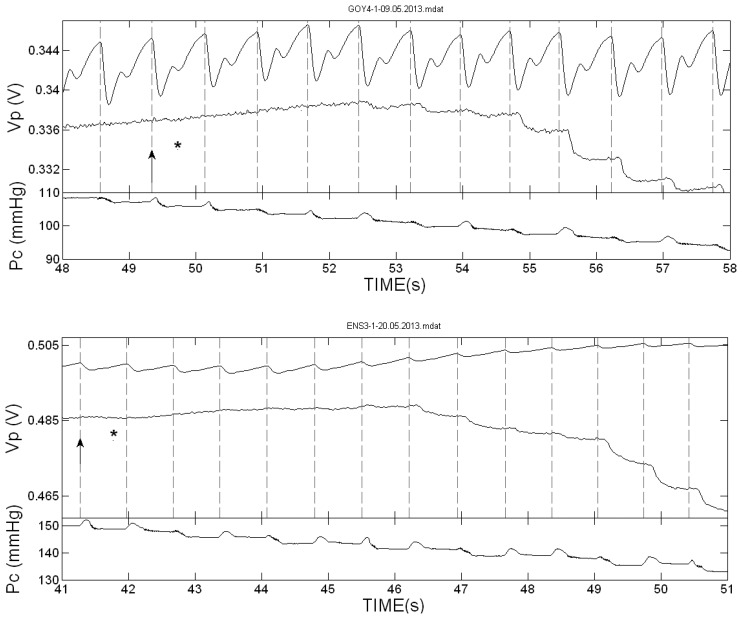
The PPG pulses (V_P_) in the two hands of two of the subjects during cuff deflation for cuff pressures in the neighborhood of the SBP value, and the cuff-pressure P_c_. As in Figure 2, the star indicates the time of the first detection of the Korotkoff sounds and the arrow indicates the start time of the free-hand PPG-pulse in which the first star appeared. In these examinations, the first Korotkoff sound (indicated by a star) was heard several pulses before the PPG pulses reappeared. In these examinations Korotkoff sounds technique is more reliable than the PPG technique.

**Figure 4.**
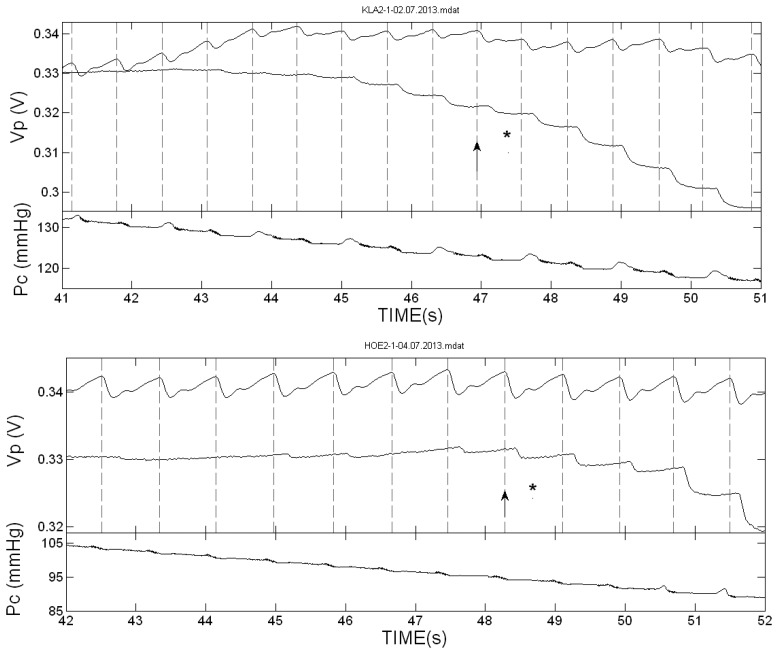
The PPG pulses in the two hands of two of the subjects during cuff deflation for cuff pressures in the neighborhood of the SBP value, and the cuff-pressure P_c_. As in Figure 2, the star indicates the time of the first detection of the Korotkoff sounds and the arrow indicates the start time of the free-hand PPG-pulse in which the first star appeared. In these examinations, the PPG pulses reappeared several pulses before the detection of the first Korotkoff sound (indicated by the star). In these examinations the PPG technique is more reliable than Korotkoff sounds technique.

The Korotkoff sounds-based SBP value (SBP_K_) was determined off-line by the indications (stars) marked by the two investigators. In order to compensate for the reaction time of the investigators (of about 1/3 s), the SBP_K_ value was determined as the cuff air pressure value, P_C_, at the start time of the free-hand PPG-pulse in which the first star appeared (see Figures 2,3 and 4 in the Results Section). In most cases the two investigators indicated their first stars in the same PPG pulse, but in some examinations the two indications differed by one or two pulses. In these cases the SBP_K_ value was determined by the first star-indication to appear, corresponding to a higher value of SBP_K_, on the assumption that the difference was either due to a difference in the hearing acuity of the investigators or to a momentary lapse of attention on the part of one of the investigators.

The PPG-based SBP value (SBP_PPG_) in each examination was obtained off-line by two observers (SE and EH) from the value of the cuff air pressure, P_C_, at which the PPG pulses reappeared during the deflation period. The two observers were blind to the corresponding pressure values determined by auscultation. At cuff pressures slightly below the SBP value, the artery is only open for a short time—when the arterial blood pressure is higher than P_C_—and the corresponding PPG pulses are smaller than the corresponding pulses in the cuff-free hand. However the characteristic pattern of these PPG pulses was distinct from the noise in most cases, and the first pulse was easily recognized. The criterion used in our study to determine the reappearance of the first PPG pulse was qualitative as is the criterion for the Korotkoff sounds. Only pulses whose appearance resembled the PPG pattern, that showed significant decrease starting 100–300 ms after the start time of the PPG signal in the free hand, were accepted. Only pulses that were recognized as PPG pulses by both observers were accepted. The SBP_PPG_ value was determined as the cuff air pressure value at the start time of the PPG pulse in the cuff-free hand in which the first PPG pulse had clearly reappeared.

### 2.4. Comparison of the Relative Accuracy of SBP_K_ and SBP_PPG_

In order to obtain the absolute accuracy of the indirect measurement of SBP based on a pressure cuff and on either Korotkoff sounds or PPG, comparison to invasive measurement by arterial catheter—the gold standard—is required. In the current study a method for the assessment of the relative accuracy of SBP_K_ and SBP_PPG_ was used, and this did not require invasive measurements.

In former studies we have found that in simultaneous measurements of SBP by Korotkoff sounds and PPG, similar SBP values were obtained by both techniques in the majority of examinations. In some examinations, however, differences in SBP values between the two techniques were found. Since both techniques are indirect we cannot know *a priori* which of them is more accurate, but we can reveal it in a given examination by finding the technique which first detects a pulse. When the first PPG pulses are observed during the cuff deflation, it is evident that the artery below the cuff has been reopened, and the SBP value is higher than the momentary cuff pressure. If Korotkoff sounds are not detected at that time, it is a failure of the auscultatory technique. If Korotkoff sounds are heard in a given examination while no PPG pulse is detected, it is a failure of the PPG technique, and the Korotkoff technique is more accurate than the PPG technique in this examination.

## 3. Results and Discussion

### 3.1. Results

Figure 1 presents the two PPG curves in one of the examinations, in the finger distal to the cuff and in the cuff-free hand, during the inflation and the deflation of the cuff. The cuff pressure as a function of time is also shown. During cuff inflation the baseline of the PPG signal decreased (lower light transmission) due to accumulation of blood volume distal to the cuff, which compresses the veins underneath and prevents drainage of blood from the hand. The PPG baseline decrease stopped before the cessation of the inflation, when the cuff pressure increased to above the SBP value and stopped blood flow to the hand. When the cuff pressure increased to above the SBP value, the PPG pulses distal to the cuff disappeared, and they returned when the cuff pressure decreased to below the SBP value. Simultaneously with the reappearance of the PPG pulses, the baseline of the PPG signal presented significant decrease, due to additional accumulation of blood in the finger. The stars in the figure indicate the time at which Korotkoff sounds were heard by the two listeners. Similar curves were obtained for all other examinations.

Figure 2 presents the PPG pulses in the two hands of two of the subjects during cuff deflation in the neighborhood of the SBP value. The vertical dotted lines indicate the start of the systolic decrease in the cuff-free hand PPG pulses. In these two examinations the first Korotkoff sound was heard in the same heart beat or nearly the same beat in which the first PPG pulse was detected. Figure 3 presents the PPG signals in the neighborhood of the SBP value in examinations in which the Korotkoff sounds were heard before the first PPG pulses were detected, and Figure 4 presents similar PPG curves in which PPG pulses were observed before the first Korotkoff sounds were detected.

The curves below the PPG curves are the air pressure (P_c_) curves during the cuff deflation. Note the oscillations in the decreasing pressure curves that appear before the SBP values, both those obtained by Korotkoff sounds and those obtained by the PPG pulses reappearance. These oscillations are used in oscillometry to determine the systolic and diastolic blood pressure values through empirical criteria.

**Figure 5.**
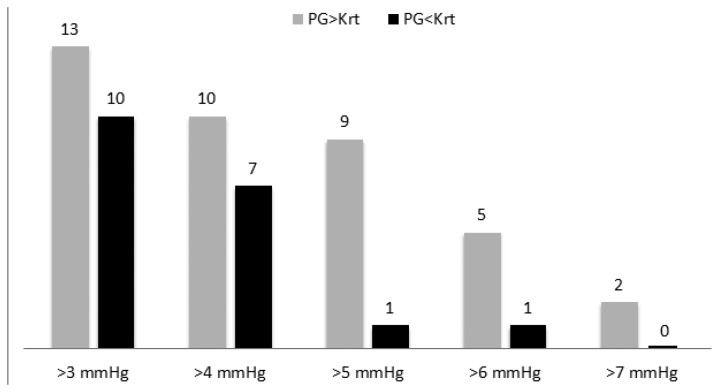
The number of examinations in which SBP_K_ and SBP_PPG_ differed by more than 3, 4, 5, 6 and 7 mmHg. SBP_PPG_ was higher than SBP_K_ in more examinations than those in which SBP_K_ was higher than SBP_PPG_.

In 97 out of 120 examinations of the 40 subjects, the difference between SBP values, measured by PPG, and SBP values measured by Korotkoff sounds was less than 3 mmHg. The mean (±SD) of the difference SBP_PPG_-SBP_K_ for the 120 examinations was 0.81 (±2.49) mmHg, which is significantly higher than zero (p < 0.001 in t-test). Figure 5 shows the number of examinations in which SBP_K_ differed from SBP_PPG_ by 3, 4, 5, 6 and 7 mmHg or more.

**Figure 6.**
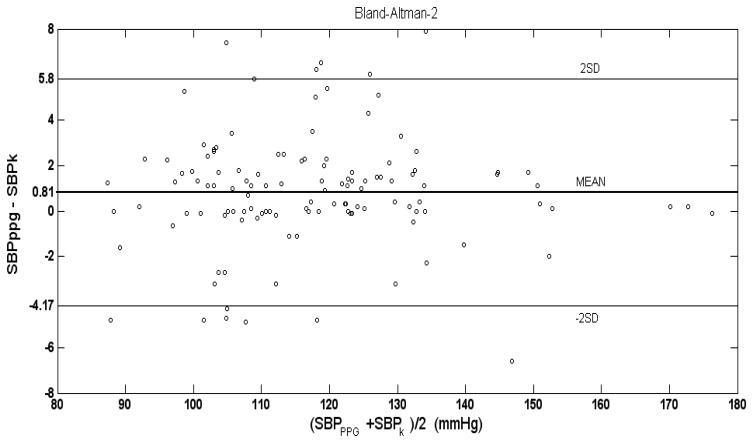
Bland-Altman plot of the difference between SBP values measured by PPG and by Korotkoff sounds (SBP_PPG_-SBP_K_) as a function of their mean. The mean difference was 0.81 mmHg and the 2SD values were ±4.98 mmHg. More examinations showed positive SBP_PPG_-SBP_K_ than negative SBP_PPG_-SBP_K_.

Figure 6 shows the Bland-Altman plot of the difference between the SBP values, measured by PPG and by Korotkoff sounds, against the inter-method average. In many of the examinations, the difference, SBP_PPG_-SBP_K_, was zero.

In the majority of the examinations in which SBP_PPG_-SBP_K_ differed from zero, SBP_PPG_ was higher than SBP_K_. As was claimed above, in a given examination in which SBP_PPG_ and SBP_K_ differed, detection of the PPG signal before the Korotkoff sounds during the cuff deflation indicates higher accuracy of the PPG-based technique relative to the Korotkoff based-technique and *vice versa*.

**Figure 7.**
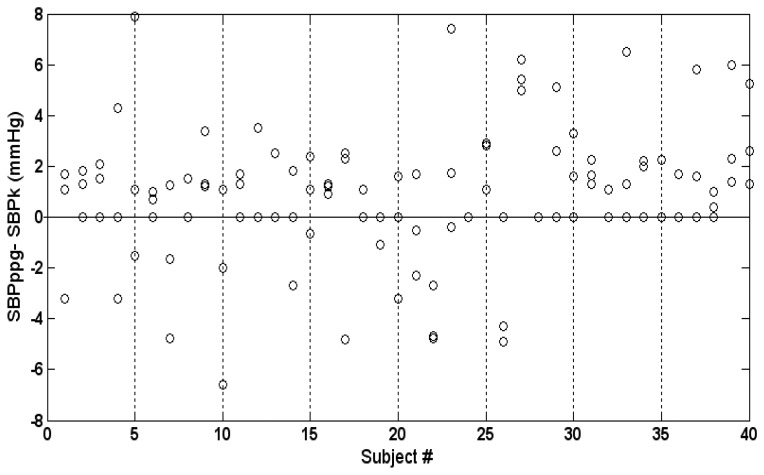
Intra-subject variability. The figure presents the three values of SBP_PPG_-SBP_K_ for each subject.

Intra-subject variability is demonstrated in Figure 7, which presents the three values of the difference SBP_PPG_-SBP_K_ for each subject. The maximal intra-subject difference between the three values of SBP_PPG_-SBP_K_ was 0–9.4 mmHg (mean and SD 3.4 ± 2.5 mmHg).

### 3.2. Discussion

The various indirect non-invasive techniques for the measurement of systolic blood pressure are based on applying decreasing external pressure by means of a pressure cuff on an underlying artery and determining the systolic blood pressure by measuring some physiological parameter that changes when the cuff pressure decreases from above the SBP value to below it. The indirect techniques assume that the counter-pressure applied on the arterial wall causes it to collapse when the cuff pressure is higher than the arterial blood pressure, and the artery reopens when the arterial blood pressure is higher than the cuff pressure. The arterial wall itself is assumed to be compliant when the trans-mural pressure is zero and to exert negligible resistance force. In stiffened atherosclerotic arteries, higher pressure than the arterial blood pressure is required to occlude the artery, and it affects blood pressure measurement [3,8,28]. This effect, pseudo-hypertension, is common to all cuff-based techniques for SBP measurements, and is not addressed in the current paper. We will discuss the physiological parameters that change when the cuff pressure decreases from above the SBP value to below it.

The cuff-based indirect techniques can be classified into three classes according to the type of these physiological parameters:

- SBP meters based on detection of a pulsatile parameter that is directly induced by blood flow and can be detected non-invasively. These pulsatile parameters include pulse palpation, ultrasound Doppler, PPG and strain gauge. SBP is determined from the cuff pressure at which the pulsatile parameter reappears when the external pressure decreases below the SBP value and the artery reopens.
- Auscultatory sphygmomanometry based on Korotkoff sounds. Korotkoff sounds are also assumed to start when the external pressure decreases below the SBP value and the artery reopens. However, they cannot be classified as directly induced by blood flow, since they are not heard when no external pressure is applied on the artery, when blood flows uninterruptedly.
- Oscillometry, which is based on the dependence on time of the air pressure pulses in the cuff during cuff deflation—the oscillometric curve. The air pressure pulses appear even when the cuff pressure is above SBP value, and the SBP is determined by the analysis of the oscillometric curve using empirical criteria. Those empirical criteria are probably responsible for the low accuracy of oscillometry [3,7–9], since the oscillometric curve changes significantly when there are alterations in arterial properties.

Korotkoff sounds appear only when the cuff pressure is between the systolic and diastolic blood pressure. The arterial blood flow itself does not generate these sounds, as they are not heard when the cuff pressure is below the diastolic blood pressure value. Several effects have been suggested as the origin for Korotkoff sounds: turbulence due to partial occlusion of the artery, flutter of the arterial wall, impact of the pulsating blood on the stationary distal column of blood and more [18,29–31]. It is reasonable to assume that several effects take part in the creation of the Korotkoff sounds.

Auscultatory sphygmomanometry, based on the detection of Korotkoff sounds, has been accepted as the preferred non-invasive technique for the measurement of arterial blood pressure. Auscultatory sphygmomanometry has been compared to direct intra-arterial measurement of SBP, and it provided, in several examinations, significantly lower values than those obtained by the invasive gold-standard [3,6,32–34]. It seems that in some examinations, when the cuff pressure is slightly below the SBP value, the effects that generate Korotkoff sounds may not be strong enough to produce audible Korotkoff sounds.

The PPG-based technique belongs to the first class, since the PPG signal can appear only when the cuff pressure decreases below the SBP value, and it continues to appear when there is no external pressure. In the current study we compared the PPG-based technique with auscultatory sphygmomanometry, which is considered to be the most accurate non-invasive method for blood pressure measurement and is accepted as the method of choice. In our study we have found that during cuff deflation, in the majority of the examinations in which SBP_PPG_ differed from SBP_K_, SBP_PPG_ was higher than SBP_K_ (see Figure 6). The mean of the difference SBP_PPG_-SBP_K_ was positive, 0.81 mmHg, and the number of examinations in which SBP_PPG_ was higher than SBP_K_ by 3, 4, 5, 6 and 7 mmHg or more (in a given examination) was higher than the corresponding number of examinations in which SBP_K_ was higher than SBP_PPG_. Detection of the PPG signal before the Korotkoff sounds during cuff deflation indicates the greater accuracy of the PPG-based technique relative to the Korotkoff based-technique in that measurement, and *vice versa*. In the current study, the PPG-based measurement of the SBP was found to be more accurate than the Korotkoff sounds-based technique of the SBP measurement. It should be emphasized that the current study cannot provide information regarding the absolute accuracy of the two techniques: in order to make such a determination, comparison to intra-arterial measurement is required.

The study also showed that in some cases the PPG-based technique did not provide an accurate SBP measurement. In those examinations in which Korotkoff sounds were heard before the reappearance of the PPG signals, the latter were not detected despite the reopened artery, as proved by the detection of the Korotkoff sounds. The small volume of blood added to the whole arm and hand during the short time in which the arterial blood pressure was above the instantaneous cuff pressure could not create a detectable change in light transmission in several examinations. It is reasonable, however, to assume that increase of the PPG signal-to-noise ratio and more reliable detection of the PPG signal can provide higher accuracy in the measurement of the SBP by the PPG-based technique. It is not reasonable to expect that after 100 years of extensive use, the Korotkoff sounds-based technique can be significantly improved.

## 4. Conclusions

The current study shows that the PPG-based technique for the measurement of SBP is of comparable accuracy to the Korotkoff sound-based technique, and probably of higher accuracy, though this last claim should be validated in more comprehensive studies. It should be emphasized that the study was performed on male subjects, who have PPG signal greater than that for female subjects. In general, the typical pattern of the PPG signals can be clearly recognized even when the cuff pressure is slightly below the SBP value, indicating that automatic measurement of SBP by means of automatic detection of the PPG signals is feasible. The absolute accuracy of the automatic measurement of the SBP by means of PPG-based technique has to be determined experimentally by comparing it to invasive intra-arterial blood pressure measurements.

The main limitation of the PPG-based technique is that it only measures SBP but not diastolic blood pressure. However, a blood pressure meter, that provides automatic and accurate measurement of SBP together with diastolic blood pressure measurement either by the manual Korotkoff sound-based technique or by the automatic oscillometry, seems to be of clinical merit.

During the long period since the invention of the Korotkoff sounds-based technique, efforts have been made to use the technique automatically, using a microphone instead of a stethoscope, but the automatic technique has not been shown to be accurate enough to replace auscultatory sphygmomanometry. Audible artifacts are more reliably rejected by the human ear-brain combination than by sophisticated algorithm designed to analyze the microphone signals and to differentiate between Korotkoff sounds and artifacts. The PPG signal seems to be more suitable for automatic detection because in most cases the pattern of the PPG signals is conserved throughout the whole deflation period and can be easily recognized.
